# Supplementary material for: Modifications in Gene Expression in the Process of Osteoblastic Differentiation of Multipotent Bone Marrow-Derived Human Mesenchymal Stem Cells Induced by a Novel Osteoinductive Porous Medical-Grade 3D-Printed Poly(ε-caprolactone)/β-tricalcium Phosphate Composite
Source: Int J Mol Sci. 2021 Oct 18;22(20):11216. doi: 10.3390/ijms222011216 (PMC8537621; doi:10.3390/ijms222011216)
Supplement: Supplementary file 1 [file ijms-22-11216-s001.zip › ijms-1402318-supplementary.pdf]

International Journal of
Molecular Sciences

MDPI

SUPPLEMENTARY INFORMATION

Article

Modifications in gene expression in the process of osteoblastic differentiation of multipotent bone marrow-derived human mesenchymal stem cells induced by a novel osteoinductive porous medical-grade 3D-printed poly(ε-caprolactone)/ β-tricalcium phosphate composite

Ivan López-González [1,*,†], Camilo Zamora-Ledezma [1,†], María Isabel Sanchez-Lorencio [2], Elena Tristante Barrenechea [3], Gabaldón J.A. [4] and Luis Meseguer-Olmo [1,*,†]

[1] Tissue Regeneration and Repair Group: Orthobiology, Biomaterials and Tissue Engineering. UCAM-Universidad Católica de Murcia, Campus de los Jerónimos 135, Guadalupe, 30107 Murcia, Spain. ilopez27@ucam.edu (I.L.-G.); lmeseguer@ucam.edu (L.M.-O); czamora9@ucam.edu (C.Z.-L.)
[2] Biomedical Research Institute of Murcia (IMIB-Arrixaca-UMU), University Clinical Hospital "Virgen de la Arrixaca", University of Murcia, El Palmar, 30120 Murcia, Spain. msl70082@um.es (MI.S.-L.)
[3] Plataforma Sala Blanca. IMIB-Arrixaca. Carretera Madrid-Cartagena S/N. El Palmar, 30120, Murcia, Spain. elena.tristante@imib.es (E.T.B)
[4] Molecular Recognition and Encapsulation Research Group (REM), Health Sciences Department, UCAM-Universidad Católica de Murcia, Campus de los Jerónimos 135, Guadalupe, 30107 Murcia, Spain; jagabaldon@ucam.edu (G.J.A.)
* Correspondence: ilopez27@ucam.edu (I.L.-G.), lmeseguer@ucam.edu (L.M.-O); Tel.: +34- 968-278800
† These authors contributed equally to this work

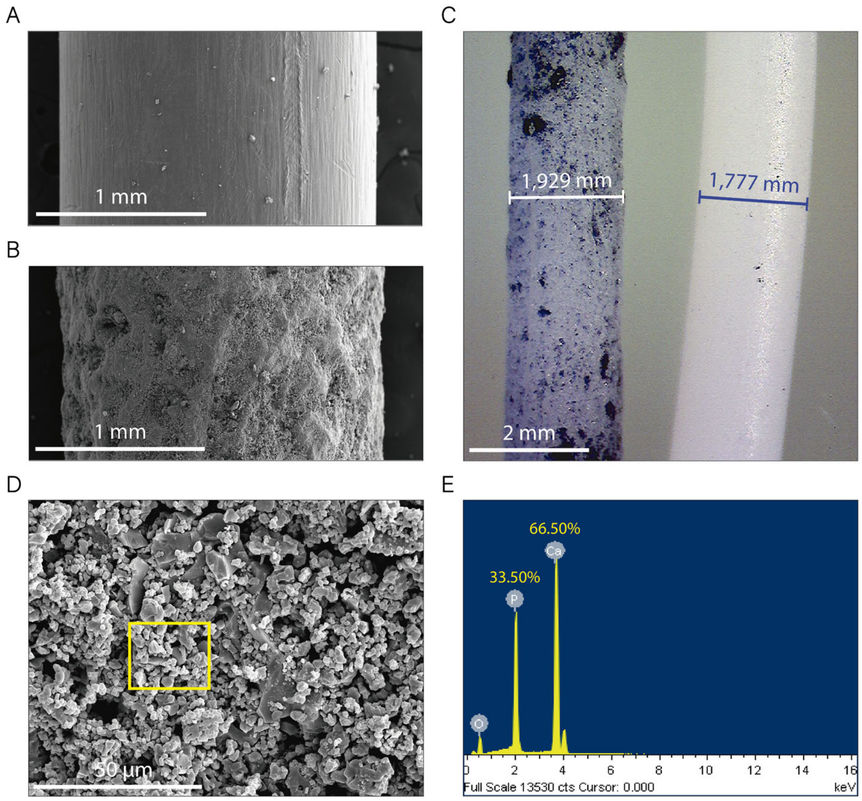

Figure S1. SEM micrographs of the (A) native PCL and (B) PCL/β-TCP filaments. (C) Image of both filaments taken with a stereomicroscope at 3X magnification. The β-TCP microparticles were stained with nigrosine to get clear and highly defined images. (D) Micrograph showing a magnification of the β-TCP microparticles coating the filament and (E) its corresponding EDX spectrum. The researched area is depicted as a yellow square
